# Solution combustion synthesis of Ni/La_2_O_3_ for dry reforming of methane: tuning the basicity *via* alkali and alkaline earth metal oxide promoters†

**DOI:** 10.1039/d1ra05511a

**Published:** 2021-10-15

**Authors:** Yahia H. Ahmad, Assem T. Mohamed, A. Kumar, Siham Y. Al-Qaradawi

**Affiliations:** Department of Chemistry and Earth Sciences, College of Arts and Sciences, Qatar University Doha 2713 Qatar siham@qu.edu.qa; Department of Chemical Engineering, College of Engineering, Qatar University Doha 2713 Qatar

## Abstract

The production of syngas *via* dry reforming of methane (DRM) has drawn tremendous research interest, ascribed to its remarkable economic and environmental impacts. Herein, we report the synthesis of K, Na, Cs, Li, and Mg-promoted Ni/La_2_O_3_ using solution combustion synthesis (SCS). The properties of the catalysts were determined by N_2_ physisorption experiments, scanning electron microscopy (SEM), transmission electron microscopy (TEM), X-ray diffraction (XRD), X-ray photoelectron spectrometry (XPS), and H_2_-TPR (temperature programmed reduction). In addition, their catalytic performance towards DRM was evaluated at 700 °C. The results demonstrated that all catalysts exhibited porous structures with high specific surface area, in particular, Mg-promoted Ni/La_2_O_3_ (Mg–Ni–La_2_O_3_) which depicted the highest surface area and highest pore volume (54.2 m^2^ g^−1^, 0.36 cm^3^ g^−1^). Furthermore, Mg–Ni–La_2_O_3_ exhibited outstanding catalytic performance in terms of activity and chemical stability compared to its counterparts. For instance, at a gas hourly space velocity (GHSV) of 30 000 mL g^−1^ h^−1^, it afforded 83.2% methane conversion and 90.8% CO_2_ conversion at 700 °C with no detectable carbon deposition over an operating period of 100 h. The superb DRM catalytic performance of Mg–Ni–La_2_O_3_ was attributed to the high specific surface area/porosity, strong metal-support interaction (MSI), and enhanced basicity, in particular the strong basic sites compared to other promoted catalysts. These factors remarkably enhance the catalytic performance and foster resistance to coke deposition.

## Abstract 2

Alkali and alkaline earth metal oxides-promoted Ni/La_2_O_3_ catalysts synthesized by solution combustion synthesis revealed enhanced catalytic performance towards dry reforming of methane.

## Graphical abstract

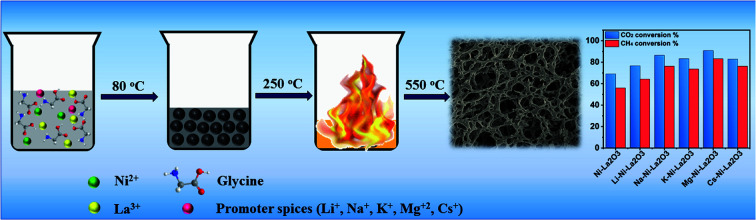

## 1. Introduction

The dramatic increment in energy demand and rapid depletion of fossil fuels have spurred remarkable efforts towards green alternative energy sources. One of the dedicated approaches to address these issues is the dry reforming of methane (DRM) in which two greenhouse gases *i.e.* carbon dioxide and methane are converted to syngas (CO + H_2_), the feedstock for synthesis of methanol and other value-added chemicals. DRM is a highly endothermic reaction that becomes spontaneous at 640 °C.^1^ DRM can be catalysed by noble elements such as Rh, Ir, Pt, Pd, and Ru, which afford outstanding catalytic activity and stability.^2–6^ However, their high cost and scarcity prohibit wide-scale adoption.^7^ Several transition metals such as Co, Ni, and Fe have been suggested as substitutes for noble metals. Among them, Ni has drawn remarkable interest owing to its low cost and high catalytic activity. However, the deactivation of active sites induced by high temperature sintering and extensive deposition of coke are considered as the main stumbling blocks towards commercialization.^8^ Different strategies were employed to improve the performance of Ni-based catalysts towards DRM, such as alloying of Ni with other metals,^9^ addition of promoters,^10^ and confinement of Ni into mesoporous support.^11^

According to previous reports, there is a consensus view that metal-support interaction (MSI) remarkably influence the catalytic performance of the DRM catalyst. Based on this, different metal oxides have been hired as supports for Ni-based DRM catalysts such as SiO_2_,^12^ Al_2_O_3_,^13^ TiO_2_,^11^ CeO_2_,^14^ ZrO_2_,^15^ and La_2_O_3_.^12^ Among them, La_2_O_3_ received great interest ascribed to its parallel function as support and promoter, where it is capable to afford basic sites that enhance the adsorption of CO_2_*via* formation of La_2_O_2_CO_3_. This endows the removal of deposited coke through the following reaction: La_2_O_2_CO_3_ + C → La_2_O_3_ + 2CO.^16,17^ Concurrently, La_2_O_3_ enables strong interaction with Ni which promotes high dispersion of Ni particles, reduces the Ni particle size, and prevents the high temperature sintering.^18^

The addition of basic promoters, in particular, the alkaline earth metals oxides remarkably enhance the catalytic performance of Ni-based catalysts.^19^ Among these promoters, MgO received great interest attributed to its remarkable catalytic performance and enhanced coking resistance.^10^ For example, the catalytic performance of Ni-supported Mg–La mixed oxides (with different La^3+^/Mg^2+^ ratios) towards DRM was studied.^20^ Activity and stability measurements depicted that Ni/10MgO–La_2_O_3_ afforded the best performance and mainly forms monoclinic La_2_O_2_CO_3_, which enhanced the coke removal contrarily to the hexagonal phase, which has no impact on the catalytic activity.^20^ In another work, the DRM performance of MgO-promoted Ni/Al_2_O_3_ catalyst synthesized by loading of MgO on Ni/Al_2_O_3_ prepared by atomic layer deposition of Ni on Al_2_O_3_ nanoparticles was studied.^21^ It was found that the addition of MgO enhanced the amount and strength of the catalyst basic sites, and increase the intensity of surface oxygenated species that enhance the adsorption and activation of CO_2_. In addition, MgO promoted the resistance against coke formation, in particular, graphitic carbon, which is responsible for catalyst deactivation.^21^ Although the promotion of Ni/La_2_O_3_ with alkaline earth metals was previously investigated in the literature, however, the impact of alkali metals was not emphasized enough.

In terms of the synthesis approach, solution combustion synthesis (SCS) received increased interest as it permits the synthesis of wide range of materials in nanoscale dimensions such as metal oxides, sulfides, phosphates, metals and alloys.^22^ SCS allows the controlling of size, composition, and nanoarchitecture of materials through self-sustained exothermic reactions. This synthesis approach endows several merits such as simplicity, low cost, high porosity of synthesized materials, and small particle size.^23^ Nevertheless, several preparation routes were reported for the synthesis of Ni/La_2_O_3_ catalysts such as incipient wetness impregnation method^24^ and sol–gel approach,^25^ whereas its synthesis *via* solution combustion procedure was not previously investigated.

Triggered by the above discussions, herein, we introduce the synthesis of alkali metals oxides and MgO-promoted Ni/La_2_O_3_ using SCS for DRM. The freshly reduced samples as well as spent samples (after DRM operation) were investigated using different structural techniques. Besides, the catalytic activity and stability of studied catalysts was evaluated at 700 °C.

## 2. Experimental

### 2.1. Materials and reagents

Nickel nitrate hexahydrate (≥98.5%), sodium nitrate (≥98.5%), magnesium nitrate hexahydrate (≥99.0%), lithium nitrate, cesium nitrate (99.0%), potassium nitrate (99.0%), and glycine (≥98.5%) were purchased from Sigma–Aldrich. Lanthanum nitrate hexahydrate (99.99%) was purchased from Carl-Roth, Germany.

### 2.2. Catalyst synthesis

#### 2.2.1. Synthesis of Ni–La_2_O_3_

To a beaker containing 50 mL DI H_2_O, (0.291 g, 1 mmol) of nickel nitrate hexahydrate, (2.165 g, 5 mmol) of lanthanum nitrate hexahydrate, and (0.788 g, 10.5 mmol) of glycine were added. The mixture was stirred for 60 minutes until it became homogeneous. Afterwards, the solution was heated at 250 °C until complete dryness, ignition, and combustion took place. The as-formed solid was collected and calcined in air at 550 °C for 2 h at a ramp rate of 1 °C min^−1^.

#### 2.2.2. Synthesis of X–Ni–La_2_O_3_ (X = Cs, Li, K, Na, or Mg)

The promoted Ni–La_2_O_3_ was synthesized with the same procedure of Ni–La_2_O_3_ with addition of 0.5 mmol of the nitrate precursor of the promoting species.

### 2.3. Catalyst characterization

The catalysts morphology was investigated by field emission scanning electron microscopy (FESEM) using Philips XL-30 microscope. The morphology of reduced catalysts was investigated using bright field transmission electron microscopy (TEM) *via* FEI Tecnai G2 TF20 UT microscope at an accelerating voltage of 200 kV. The samples composition was investigated by inductively coupled plasma optical emission spectrometry (ICP-OES) *via* a spectrometer (PerkinElmer, Optima 5300 DV). The textural properties were examined *via* N_2_ sorption experiments at liquid nitrogen temperature (77 K) using the Brunauer–Emmett–Teller (BET) method. X-ray diffraction (XRD) spectra was recorded using X'Pert-Pro MPD diffractometer (PANalytical Co., Netherlands) using of Cu-Kα X-ray source (*λ* = 1.54059 Å) as a radiation source in the 2*θ* range (10–80). The chemical composition and the elemental oxidation states were investigated using XPS spectrophotometer Kratos Axis Ultra XPS equipped with a monochromatic Al-Kα radiation source (1486.6 eV) under ultra-high vacuum (UHV) (*ca.* 5 × 10^−9^ torr).

The thermogravimetric analysis was executed using TGA 4000 analyzer (PerkinElmer, USA). Measurements were performed at the temperature range 25–850 °C at a heating rate of 10 °C min^−1^ under air flow. Samples reducibility was examined using temperature-programmed reduction of hydrogen (H_2_-TPR). Measurements were carried out with Micromeritics Autochem 2920 chemisorption analyzer with H_2_ uptake recorded *via* TCD detector. 50 mg of the supported catalyst was placed into a quartz U-shape tube and 10% H_2_ balanced in Ar was passed over the test sample in a flow rate of 10 mL min^−1^ until a stable baseline is attained. The sample was then heated in a ramp rate of 5 °C min^−1^ in the temperature range 30–850 °C. The basicity was examined by CO_2_-TPD. 50 mg of the sample was first reduced in a flow of 10% H_2_ at 800 °C for 2 h, then the ample was cooled down to room temperature. Then, the reduced sample was degassed at 300 °C in a flow of Ar for 1 h followed by cooling to 50 °C. Afterwards, 10% CO_2_ is injected at a rate of 30 mL min^−1^ for 1 h at 50 °C followed by purging of Ar to remove excess CO_2_, then the sample was heated under Ar flow at a ramp rate 10 °C min^−1^ to 900 °C and the signal of desorbed CO_2_ is detected by TCD.

### 2.4. DRM activity

DRM activity measurements were carried out in a fixed bed quartz tube reactor (*d* = 6 mm) at atmospheric pressure. 100 mg of the catalyst was loaded and fixed by quartz wool. Reactor temperature was recorded *via* K-type thermocouple located at the fixed bed. The catalyst was Pre-reduced with 10% H_2_ flow at 800 °C for 2 h. After which, N_2_ was purged to discard excess H_2_. The feed gas composition was (10% CH_4_ and 10% CO_2_ balanced with Ar) and was fed to the reactor in a rate of 50 mL min^−1^ giving rise to a weight hourly space velocity (WHSV) of 30 000 mL g^−1^ h^−1^. The composition of the outlet gas was investigated *via* a gas chromatograph (Agilent 7890B, Agilent Technologies, USA) coupled to a thermal conductivity detector (TCD). The catalytic performance was evaluated based on the change in the concentrations of CH_4_, CO_2_ in the inlet and outlet flow mixtures according to:
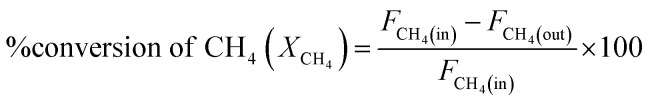

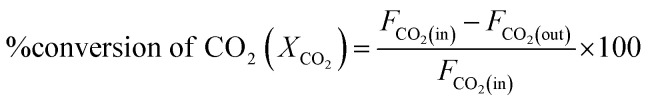

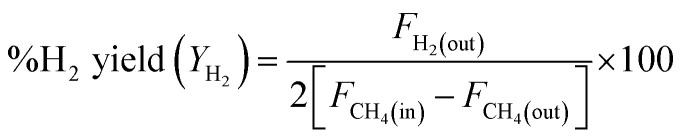

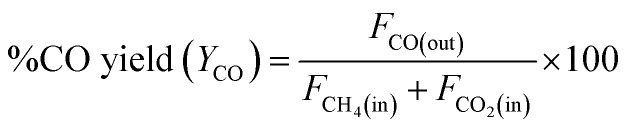

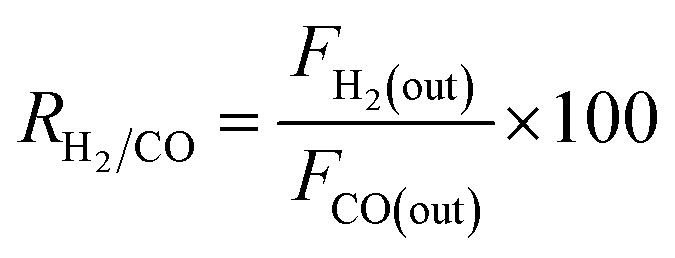
where *F*_i(in)_ and *F*_i(out)_ are the molar flow rate of inlet and outlet of species i, respectively.

## 3. Results and discussions

**Fig. 1:**
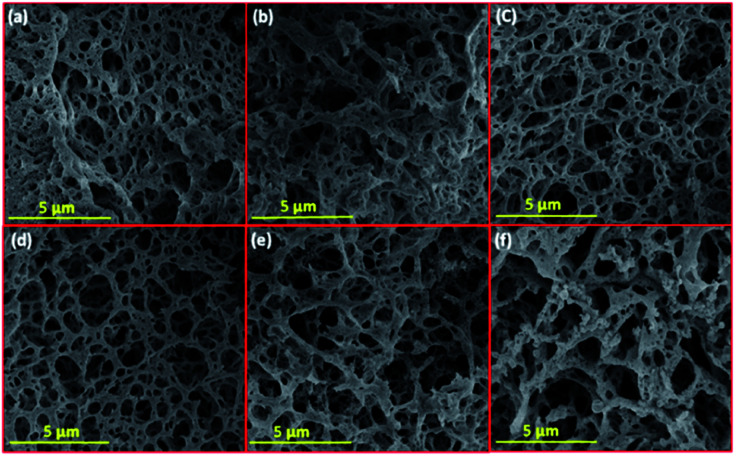
SEM images of fresh reduced samples; (a) Li–Ni–La_2_O_3_, (b) Na–Ni–La_2_O_3_, (c) K–Ni–La_2_O_3_, (d) Mg–Ni–La_2_O_3_, (e) Cs–Ni–La_2_O_3_, and (f) Ni–La_2_O_3_.

Un-promoted and promoted Ni–La_2_O_3_ were prepared using one-pot solution combustion synthesis. This procedure has several structural merits such as simplicity, energy effectiveness, and uniform distribution of components into the material's matrix, facile control of composition *via* change of oxidizer-to-fuel ratio.^26^ The Ni content of as-prepared catalysts was investigated by ICP-OES. The wt% of Ni showed comparable values ranging between 6.63–7.14% (Table 1). The morphology of as-synthesized catalysts was explored by SEM (Fig. 1).

**Table 1:** Structural properties of the investigated catalysts

Catalyst	wt% of Ni	Specific surface area *S*_BET_ (m^2^ g^−1^)	Pore volume (cm^3^ g^−1^)	Pore radius (nm)	Ni average particle size^a^ (nm)	Crystallite size^b^ (nm)
Ni–La_2_O_3_	7.14	37.3	0.18	3.7	10.3	10.8
Cs–Ni–La_2_O_3_	6.94	32.9	0.15	2.1	13.4	14.0
K–Ni–La_2_O_3_	6.69	29.7	0.16	2.1	11.6	12.1
Li–Ni–La_2_O_3_	6.63	42.9	0.24	2.5	14.3	14.9
Mg–Ni–La_2_O_3_	6.78	54.5	0.36	2.8	14.8	15.6
Na–Ni–La_2_O_3_	6.95	26.8	0.13	2.2	13.0	13.6

a Calculated from TEM.

b Calculated from XRD.

All samples depict low-density spongy-like morphology with remarkable porous architecture containing large density of voids. This morphology can be attributed to the liberation of gases within short duration during the combustion process. Samples show similar morphology except Mg–Ni–La_2_O_3_, which exhibited higher density of voids with smaller size, which is an indication to higher porosity compared to other catalysts.

**Fig. 2:**
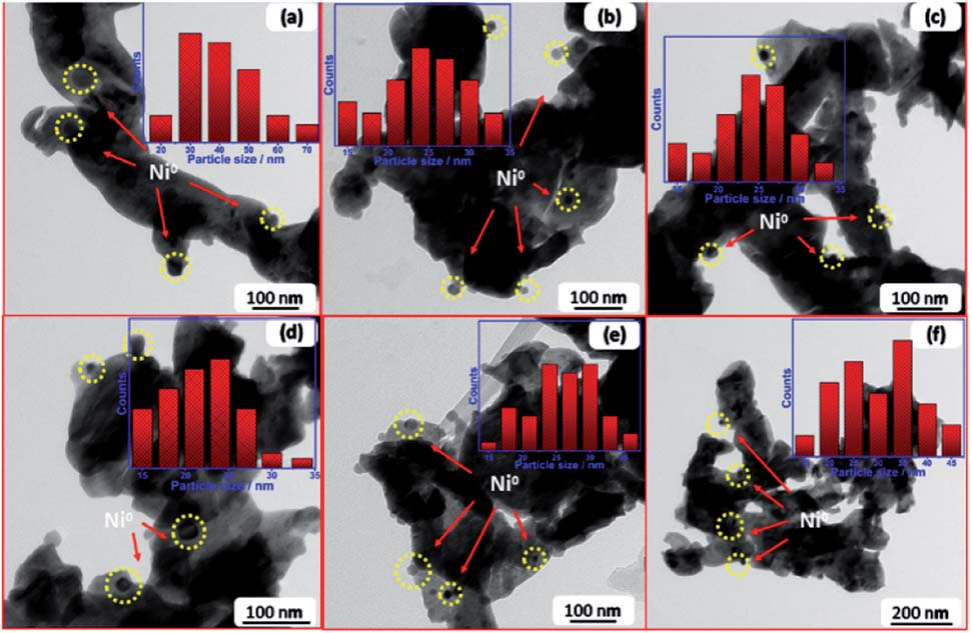
TEM images and particle size distribution of reduced samples; (a) Li–Ni–La_2_O_3_, (b) Na–Ni–La_2_O_3_, (c) K–Ni–La_2_O_3_, (d) Mg–Ni–La_2_O_3_, (e) Cs–Ni–La_2_O_3_, and (f) Ni–La_2_O_3_.

TEM of freshly reduced samples was investigated to get more deep insights on the structural properties of catalysts. The TEM images of reduced samples are shown in Fig. 2. They demonstrate good dispersion of Ni nanoparticles in the matrix of support, whereas the promoter nanoparticles cannot be detected owing to high dispersion within the support. The particle size distribution of Ni (Fig. 2 inlets) was estimated in all cases and the average size is given in Table 1. Results depicted that the incorporation of promoter did not enhance the dispersion of Ni in the La_2_O_3_ matrix, so far, the particle size of Ni increases into the promoted samples compared to pristine Ni–La_2_O_3_. This may be attributed to partial weakening in metal-support interaction encountered by the insertion of promoter into the La_2_O_3_ matrix.^27^

**Fig. 3:**
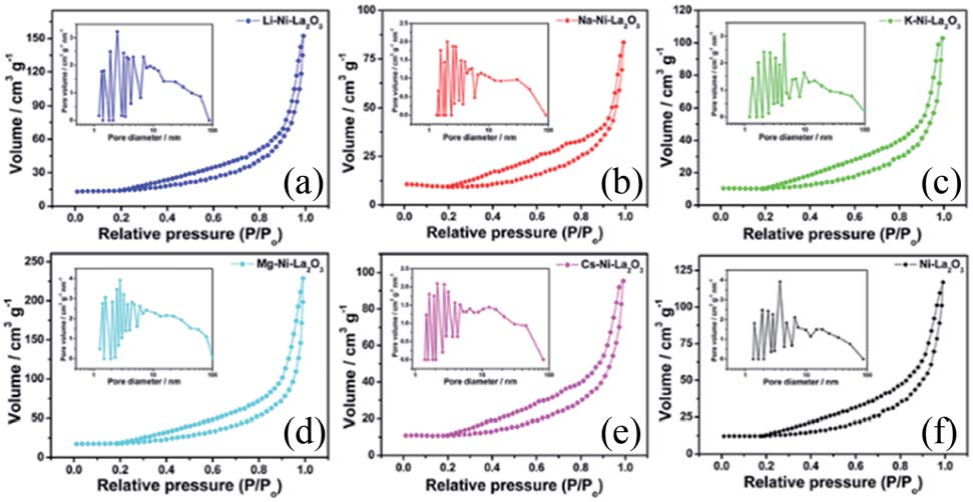
N_2_ adsorption/desorption isotherms and pore size distribution (inlet) of (a) Li–Ni–La_2_O_3_, (b) Na–Ni–La_2_O_3_, (c) K–Ni–La_2_O_3_, (d) Mg–Ni–La_2_O_3_, (e) Cs–Ni–La_2_O_3_, and (f) Ni–La_2_O_3_.

Fig. 3 displayed N_2_ adsorption–desorption isotherms and pore size distributions of investigated Ni-based catalysts. According to the International Union of Pure and Applied Chemistry (IUPAC) classification, all catalysts exhibited type IV isotherms with H3 type hysteresis loop, which is characteristic feature for mesoporous materials.^28^ Pristine Ni–La_2_O_3_ and promoted-catalysts exhibited similar shape of the isotherm with a hysteresis loop extending from over a wide range of relative pressure *P*/*P*° of 0.2 to 1, which implies a non-uniformity of pore structure and a wide pore size distribution. The similarity in textural properties between Ni–La_2_O_3_ and X–Ni–La_2_O_3_ reflects that the addition of promoter has no remarkable impact of the porous structure.^29^ The specific surface area and pore dimensions (pore volume and pore diameter) were calculated using BJH (Barrett, Joyner, and Halenda) method and the data are given in Table 1. According to the obtained results, Mg–Ni–La_2_O_3_ exhibited the highest surface area and highest pore volume compared to other samples. This means that a greater number of accessible active sites available for the reactants, which can enhance the catalytic activity of Mg–Ni–La_2_O_3_ compared to other catalysts.

**Fig. 4:**
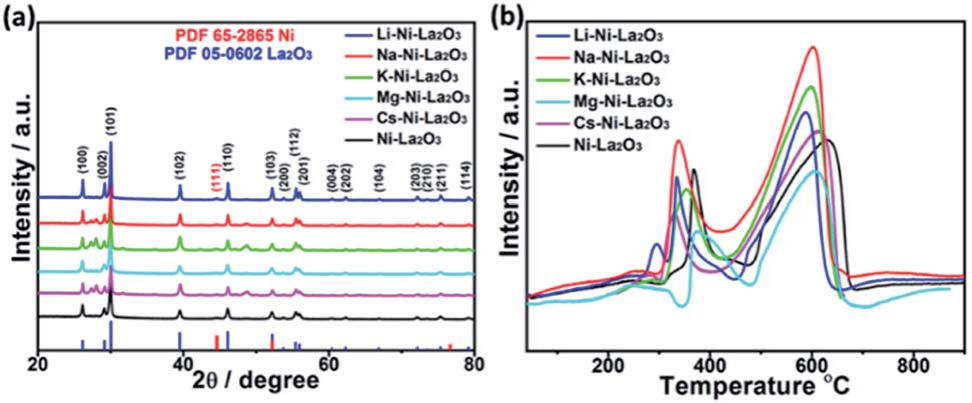
(a) XRD diffraction patterns of reduced catalysts and (b) H_2_-TPR profiles of as-prepared catalysts.

Fig. 4a represents wide-angle XRD diffraction patterns of promoted and un-promoted Ni–La_2_O_3_ after reduction in H_2_ at 800 °C for 2 h. All samples revealed the presence of the diffractions at 2*θ* of 26.1, 29.1, 30.0, 39.5, 46.1, 52.2, 53.7, 55.4, 56.0, 60.4, 62.3, 66.8, 72.2, 73.5, 75.3, and 79.2, which are corresponding to (100), (002), (101), (102), (110), (103), (200), (112), (201), (004), (202), (104), (203), (210), (211), and (114) which are the characteristic peaks of La_2_O_3_ (JCPDS card no. 05-0602), respectively. In addition, all samples exhibited a small diffraction peak at about 44.5°, corresponding to (111) diffraction of metallic Ni (JCPDS card no. 65-2865). No additional peaks were observed in the XRD patterns of promoted catalysts compared to un-promoted Ni–La_2_O_3_. This affirms that promoters have very small particle size and/or high dispersion in the La_2_O_3_ matrix, which is consistent with previous studies.^30^ The average crystallite size of different catalysts was estimated by Scherrer's equation using Ni(111) peak. The calculated values are given in Table 1. The results show that incorporation of promoters slightly increases Ni crystallite size, which is consistent with the data obtained from TEM.

Samples reducibility and redox properties of freshly prepared materials was probed by H_2_-TPR. Fig. 4b depicts the H_2_-TPR profiles of as-prepared Ni–La_2_O_3_ and promoted Ni–La_2_O_3_ samples. Similar reduction profiles were obtained for un-promoted and promoted samples (except Li–Ni–La_2_O_3_). Excluding Li-promoted catalyst, all other samples depicted two reduction peaks; the first peak in the temperature range of 320–410 °C, which is corresponding to weakly interacting NiO.^31,32^ Whereas, the second peak observed above 600 °C can be assigned to strongly interacting NiO in the form of perovskite.^33,34^ Li–Ni/La_2_O_3_ demonstrated three reduction peaks in the H_2_-TPR profile, the first peak can be attributed to the reduction of surface weakly interacting NiO species, the second peak can be assigned to bulk NiO, and the third peak corresponds to the reduction of strongly interacting NiO species in the form of perovskite. Furthermore, the lithium-promoted sample exhibits low reduction temperatures compared to other samples. The different behavior encountered by Li–Ni–La_2_O_3_ may arise from that Li_2_O remarkably weakens the interaction between Ni and La_2_O_3_, which is consistent to previous studies.^35,36^ Compared to un-promoted Ni–La_2_O_3_, all promoted samples reveal a negative shift in the reduction temperatures. This is consistent to the previous studies, which implies the same trend.^37^ This was attributed to competition between Ni and promoter species on the interaction with La_2_O_3_ support.^37^ This means that the insertion of promoter slightly decreases the interaction of Ni with La_2_O_3_ and shift the reduction of Ni species towards lower temperatures.^38^ Compared to other promoted catalysts, Mg–Ni–La_2_O_3_ demonstrated higher reduction temperatures, which affirms stronger MSI.

**Fig. 5:**
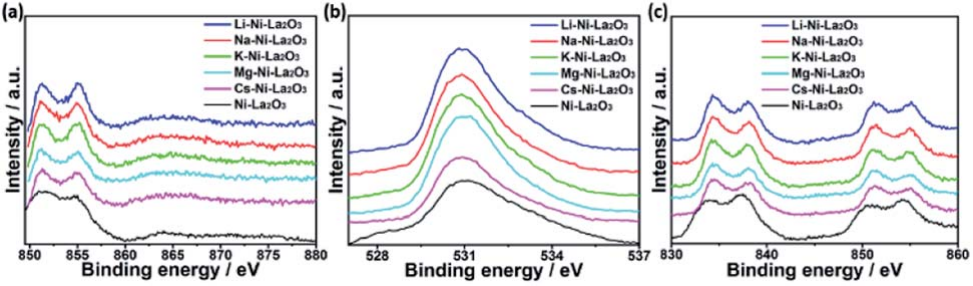
High resolution XPS spectra of (a) Ni 2p, (b) O 1s, and (c) La 3d in reduced samples.

The surface composition and chemical entities of the freshly reduced catalysts were explored by XPS analysis. The XPS survey spectra of reduced catalysts depicted the presence of the promoter species in each case together with La, Ni, and O, which confirms their chemical composition (see Fig. 5 and S1†). The binding energies of Ni, La, and O were not significantly which confirms that their electronic environment was not affected by modification with the promoters (Fig. 5a–c).

**Fig. 6:**
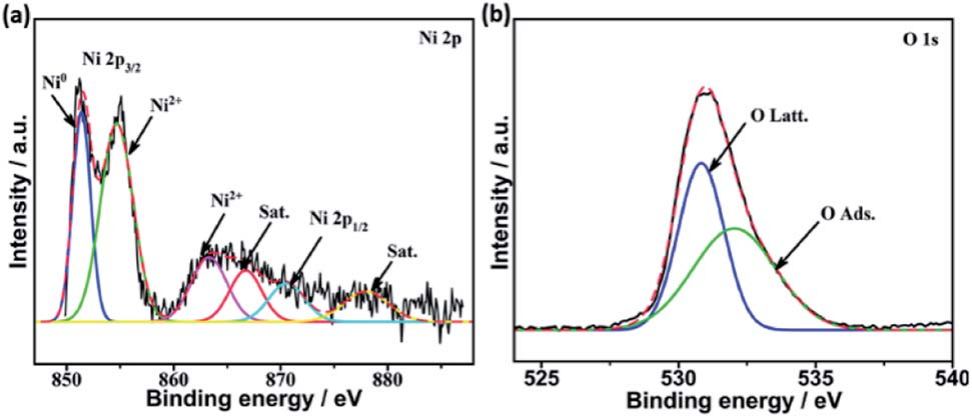
Deconvoluted high resolution XPS spectra of (a) Ni 2p and (b) O 1s in reduced Mg–Ni–La_2_O_3_.

Fig. 6a demonstrates the high resolution spectra of Ni 2p in the reduced Mg–Ni–La_2_O_3_ catalyst. Deconvolution of Ni 2p region depicted two components. The low binding energy components at about 852.4 eV is assigned to metallic Ni and the other at higher binding energy 856.3 eV corresponds to NiO.^39^ The values of binding energies are relatively higher than normal values, which implies strong interaction between Ni and La_2_O_3_ support.^40^ Deconvoluted high resolution spectra of O 1 s region manifested two peaks. The first peak at 530.4–531 eV is attributed to lattice oxygen, whereas, the other peak at about 532.5 eV can be assigned to adsorbed oxygen species such as hydroxyls and carbonates.^41^

**Fig. 7:**
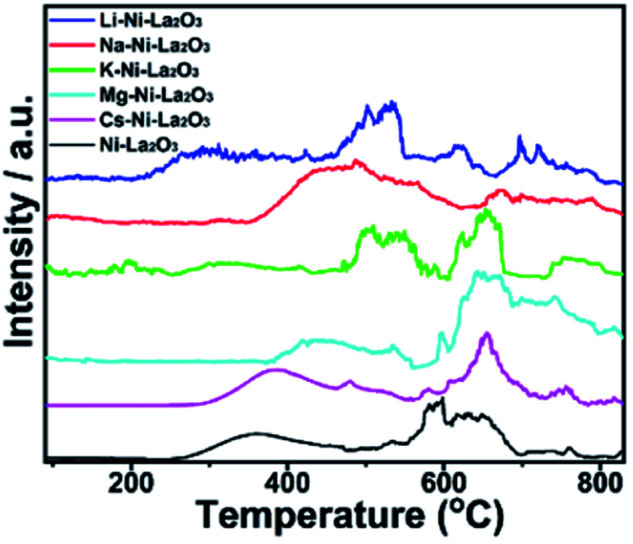
CO_2_-TPD profiles of investigated catalysts.

The basicity of samples was investigated by CO_2_-TPD. The desorption profiles are shown in Fig. 7. Three sets of peaks were observed. The desorption peaks below 400 °C can be attributed to weakly basic sites, the peaks 400–600 °C are assigned to basic sites of moderate strength, and the at temperatures higher than 600 °C corresponds to strong basic sites.^42,43^ The total amount of desorbed CO_2_ is larger in Mg–Ni–La_2_O_3_ compared to other catalysts, especially CO_2_ desorbed from strong basic sites. This implies greater amount and enhanced strength of basic sites which can facilitate the adsorption and activation of CO_2_ and enhance the DRM activity.

**Fig. 8:**
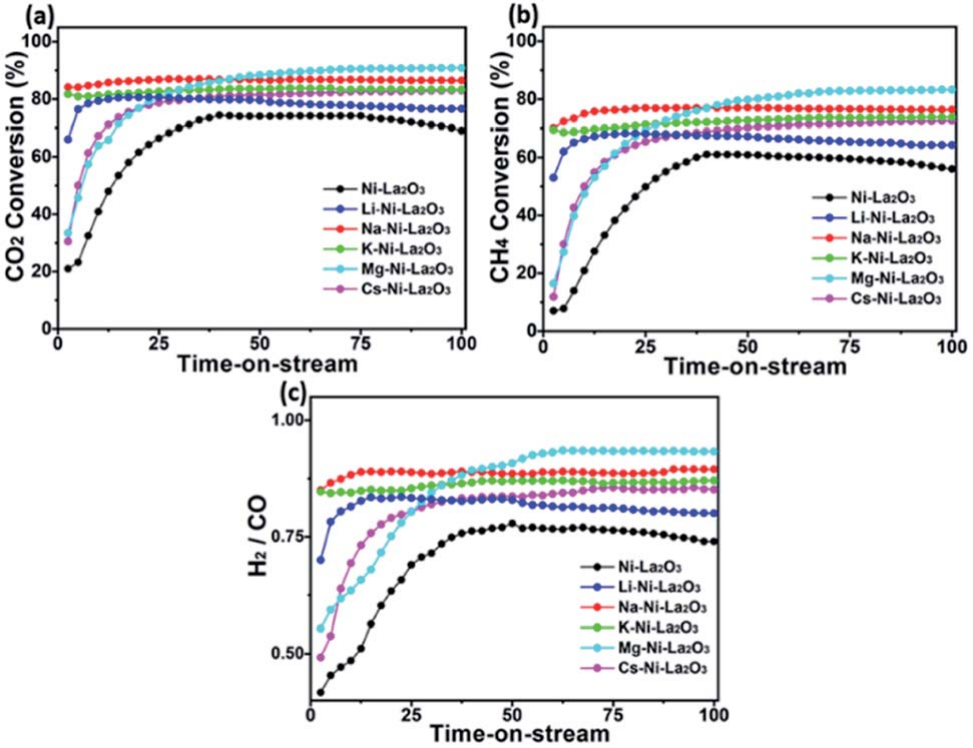
DRM catalytic performance measurements; (a) carbon dioxide conversion, (b) methane conversion, and (c) H_2_/CO ratio measured at 700 °C.

To investigate the effect of promoter type on the catalytic performance of Ni–La_2_O_3_, the DRM activity of investigated samples was examined at 700 °C for an operating time of 100 h using GHSV of 30 L g^−1^ h^−1^. Prior to the reaction, samples were reduced by 10% H_2_/Ar at 800 °C for 2 h to convert Ni species to active metallic Ni. After that, the gas mixture was purged through the fixed bed reactor and the catalytic activity was tested by evaluating the percentage of conversions of CO_2_ and CH_4_. Fig. 8 depicted the catalytic activity of promoted and un-promoted catalysts displayed in terms of variation of CO_2_ conversion, CH_4_ conversion, and H_2_/CO ratio with time-on-stream. The reaction was maintained for 100 h to explore long-term stability. Interestingly, all samples revealed slow increase in the conversion of CO_2_ and CH_4_ with time. According to previous reports, this increase in activity with time can be assigned to the incomplete reduction of Ni oxide species during the pre-treatment step.^44^ Other reports ascribed this enhancement during the initial conduction period to the slow establishment of equilibrium concentration of La_2_O_2_CO_3_, which is generated as an intermediate from the reaction of La_2_O_3_ with CO_2_.^20,45^ Intriguingly, all catalysts manifested low initial conversions for CH_4_ and CO_2_ followed by subsequent increase with time-on-stream until a steady state was attained which is consistent with previous studies.^46^ After this steady state CO_2_ and CH_4_ conversions remains constant until the end of operating time (100 h) with decay in the activity observed in CO_2_ and CH_4_ conversions in case of Ni–La_2_O_3_ and Li–Ni–La_2_O_3_. For all catalysts, CO_2_ conversions exhibited higher values than CH_4_ and the H_2_/CO ratio is less than unity. This can be attributed to the concurrent reverse water gas shift (RWGS), CO_2_ + H_2_ → CO + H_2_O.^47^

It is well known that the ratio of H_2_/CO is influenced by DRM as well as the co-occurring side reactions (Boudouard, RWGS, and methane decomposition).^12^ The actual ratio of H_2_/CO is affected by coexisting side reactions such as RWGS, methane decomposition, and Boudouard reaction. All catalysts displayed a H_2_/CO ratio, which is fluctuating around almost constant value (Fig. 8c). This fluctuation is attributable to the concurrent carbon deposition and carbon gasification reactions.^48^ The value of H_2_/CO for all catalysts lies between 0.8 and 0.9, which affirms that RWGS is the dominating side reaction.

All promoted catalysts exhibited higher CO_2_ and methane conversions compared to Ni/La_2_O_3_, which provides a clear evidence on the role of promoter in enhancing the catalytic activity. The maximum CO_2_ conversion follows the order: Mg–Ni–La_2_O_3_ (90.8%) > Na–Ni/La_2_O_3_ (86.9%) > K–Ni–La_2_O_3_ (83.7%) > Cs–Ni–La_2_O_3_ (83.0%) > Li–Ni–La_2_O_3_ (80.6%) > Ni–La_2_O_3_ (74.4%). Similarly, the maximum CH_4_ conversion follows the same order with values of 83.2%, 76.3%, 73.7%, 72.5%, 64.3%, and 56.0%, in case of Mg–Ni–La_2_O_3_, Na–Ni–La_2_O_3_, K–Ni–La_2_O_3_, Cs–Ni–La_2_O_3_, Li–Ni–La_2_O_3_, and Ni–La_2_O_3_, respectively. After reaching the maximum conversion, all catalysts demonstrated negligible decay in the activity until the end of DRM operating time (100 h) except Li–Ni–La_2_O_3_ and un-promoted catalyst, which revealed a significant decay in the activity with time. Li–Ni–La_2_O_3_ demonstrated 5.0 and 5.7% decay in the CO_2_ and CH_4_ conversion, respectively, while Ni/La_2_O_3_ exhibited 7.3% and 21.3% in the conversions of CO_2_ and CH_4_, respectively. This provides a clear evidence for the stabilizing impact of promoting species on the long-term stability of catalysts compared to Li–Ni–La_2_O_3_ and Ni–La_2_O_3_.

**Fig. 9:**
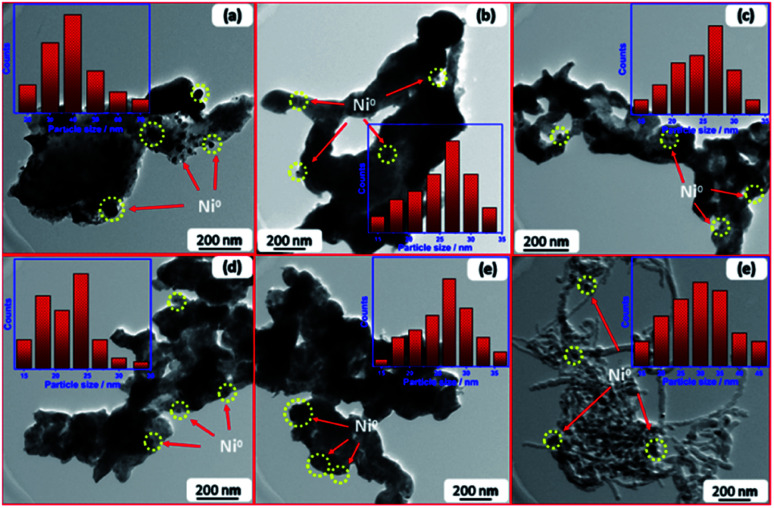
TEM images of spent samples; (a) Li–Ni–La_2_O_3_, (b) Na–Ni–La_2_O_3_, (c) K–Ni–La_2_O_3_, (d) Mg–Ni–La_2_O_3_, (e) Cs–Ni–La_2_O_3_, and (f) Ni–La_2_O_3_.

Fig. 9 displays the TEM images and Ni particle size distributions of spent catalysts after running for 100 h at 700 °C. Some Ni particles were agglomerated owing to high temperature sintering. The average particle size of Ni in the spent samples is 23.1, 30.1, 26.3, 34.2, 29.4, and 28.6 nm in case of Mg–Ni–La_2_O_3_, Na–Ni–La_2_O_3_, K–Ni–La_2_O_3_, Li–Ni–La_2_O_3_, Cs–Ni–La_2_O_3_, and Ni–La_2_O_3_, respectively. Based on the ratio of change in the Ni particle size during the reaction, Li–Ni–La_2_O_3_ and Ni–La_2_O_3_ demonstrated the greatest increase in the Ni particle size, whereas, Mg–Ni–La_2_O_3_ and Na–Ni–La_2_O_3_ revealed the lowest ratio of increase in the Ni particle size (Fig. 9). This can explain the rapid decay in the DRM activity with time in case of un-promoted and Li-promoted samples compared to other catalysts. Owing to the increase in the particle size by sintering, the number of Ni active centers decreased by agglomeration. This decreased the catalytic activity and diminished the long-term stability. This was confirmed by TEM of spent samples, which manifested the formation of filamentous type carbon in the un-promoted catalyst in contrast to all promoted samples that reveal no observable coke formation (Fig. 9).

**Fig. 10:**
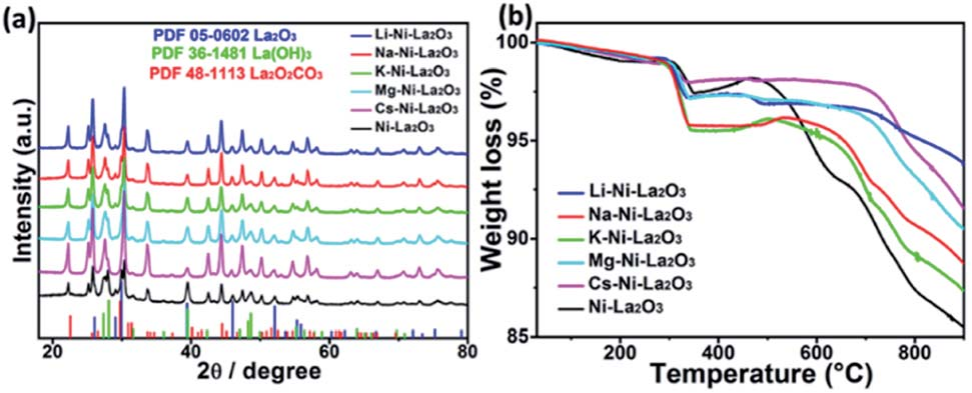
(a) XRD patterns and (b) TGA profiles of spent samples after 100 h operation time at 700 °C.

Fig. 10a depicts XRD of spent samples after DRM operating time of 100 h at 700 °C. Similar diffraction patterns were obtained by promoted Ni–La_2_O_3_ and no observable peaks were obtained for the alkali metal oxides. New diffractions peaks were observed at 2*θ* of 15.6, 27.5, 28.0, 42.5, 47.4 48.6, 50.1, 69.9, 70.9, and 75.7° corresponding to La(OH)_3_ (JCPDS card no. 36-1481). La(OH)_3_ was formed as a result of reaction between La_2_O_3_ and H_2_O that produced by RWGS *i.e.* La_2_O_3_ + 3H_2_O → 2La(OH)_3_. In addition, new diffractions were observed at 14.2, 23.1, and 29.2° which can be indexed to La_2_O_2_CO_3_ (JCPDS card no. 48-1113). During the DRM reaction, La_2_O_3_ reacts with CO_2_ to form La_2_O_2_CO_3_.^49^ Unfortunately, we couldn't calculate the Ni crystallite size in the spent samples from XRD since the Ni(111) diffraction peak overlapped with the diffractions of La_2_O_2_CO_3_ at about 44.5°. Furthermore, the graphite peak at 2*θ* of 26° could not be identified owing to the interference with (100) diffraction peak of La_2_O_3_ (JCPDS card no. 05-0602).

Fig. 10b represents the TGA profiles of spent catalysts after DRM 100 h duration time at 700 °C. The slight weight loss before 200 °C can be assigned to the release of physisorbed water in La(OH)_3_.^50^ The slight weight gain in the temperature range 300–400 °C can be ascribed to the oxidation of metallic Ni to NiO.^51^

The weight decline in the temperature range 400–600 °C owing to oxidation of coke. Only un-promoted sample revealed a noticeable weight loss in 400–600 °C, which is attributed to the oxidation of filamentous carbon, whereas, no weight loss was observed for all promoted samples in the same temperature range owing to the absence of coke deposition, which is consistent to TEM results of the spent samples. On the other hand, the mass loss above 700 °C can be attributed to the decomposition of LaO_2_CO_3_.^52^

The XPS of spent samples was investigated to probe the change in the nature and the chemical states of different elements. High resolution spectra of C 1s can provide relevant information about the nature of carbon species, respectively at the utmost surface of the spent samples. Deconvolution of C 1s spectrum results in 3 peaks at 288.4 eV, 287.5 eV and 284.6 eV which can be assigned to CO_3_^2−^, C–O, and C–C species, respectively.^44^ Whereas, the peak at lower binding energy (283.9 eV) can be assigned to Ni carbide species at the catalyst surface.^53^ The Ni 2p peak in spent samples was shifted in all catalysts towards higher binding energies assigned to NiO, implying partial decrease in the metallic character of Ni (Fig. S2a†).^54^ The differentiation between La^3+^ species in oxide and hydroxides is extremely difficult, thereon, this can be executed through the O 1s peaks.^54^ It is reported that the O 1s in La_2_O_3_ appear at lower binding energies than O 1s in La(OH)_3_. Based on this, it could be understood that La in the oxide has a greater tendency to donate electrons to the adjacent oxygen atoms compared to the La in the hydroxide. This shifted the binding energy of oxygen to lower values.^20^ This confirms that La_2_O_3_ was partially converted to La(OH)_3_ in spent samples which confirms the XRD analysis of spent catalysts (Fig. S2b†).

DRM requires bifunctional catalysts capable of promoting two simultaneous processes *i.e.* the cracking of methane and the oxidation of carbon species. It is well-known that deactivation of Ni-based catalysts during DRM is mainly assigned to the catalyst sintering as well as coke deposition.^12^ It is also noteworthy that, metal-support interaction (MSI) remarkably influence the catalytic activity and durability of the catalyst, where it enhances the stability catalyst against sintering and also against deactivation through carbon deposition. La_2_O_3_ has strong MSI with Ni catalyst, so far, it enhances Ni dispersion and stability against sintering at high reaction temperatures.^55^ It enhances the adsorption of CO_2_ to form La_2_O_2_CO_3_ which is capable of removal of deposited coke *via* the reaction: La_2_O_2_CO_3_ + 2C → La_2_O_3_ + 2CO.^56^ However, the role of MSI is not the prevailing factor affecting the catalytic performance of the investigated catalyst. This can be evident from the inferior catalytic activity and durability of un-promoted catalyst although it has higher MSI compared to promoted catalysts. Intriguingly, the particle size of Ni in fresh reduced Ni–La_2_O_3_ was smaller than all promoted samples, so far, it was expected to afford comparable catalytic performance towards DRM.^57^ However, Ni–La_2_O_3_ expressed the lowest activity and its spent catalyst showed the largest increase in the Ni particle size. Besides, only un-promoted sample manifested the formation of coke compared to all promoted samples. All these factors confirms imply that the catalyst basicity has the dominant role that affect the catalytic performance. Hence, Mg–Ni–La_2_O_3_ afforded the best catalytic activity and the highest stability.

**Scheme 1:**
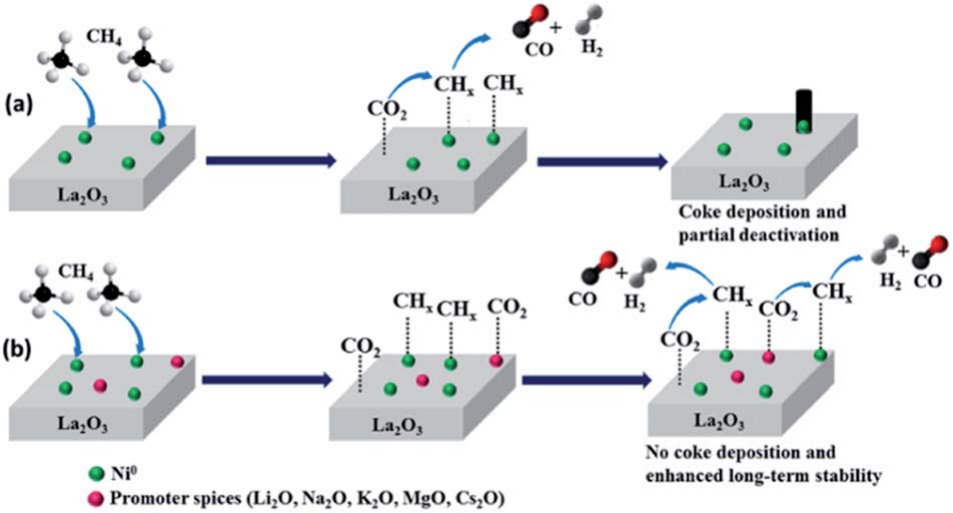
The mechanism of DRM over unpromoted and promoted catalysts.

According to the obtained results, the mechanism of DRM on un-promoted and promoted Ni–La_2_O_3_ can be represented by Scheme 1. Methane is firstly adsorbed at Ni sites and decomposed to CH_*x*_ intermediate species according to eqn (1).^58^ Simultaneously, CO_2_ is adsorbed especially at the more basic sites on La_2_O_3_ or at the promoter oxide. CH_*x*_ species are gasified according to eqn (2).^58,59^ However, the dissociation of methane on active Ni is usually a rapid process and the formed intermediate CH_*x*_ fragments may not be adjacent to activated CO_2_ molecules, which results in further growth of carbon on the active Ni centers and deposition of coke.^60^ The presence of promoter species not only enhance the basicity of La_2_O_3_ but the promoter itself can act as active sites for adsorption and activation of CO_2_. The chemisorption of CO_2_ can generate CO and active oxygen species, which enhance the gasification of CH_*x*_ species and prevent the coke deposition.^60,61^ This was affirmed by the results, which revealed coke deposition in case of un-promoted sample, whereas promoted samples do not exhibit observable deposited carbon after DRM durability tests. The MSI play an important role regarding to long-term stability, so far, Li–Ni–La_2_O_3_ exhibited weakened MSI encountered by Li_2_O, which results in sintering at high temperatures and diminishes the catalytic performance.

1 *x*CH_4_ → C_*x*_H_1−*x*_ + (5*x* − 1/2) H_2_

2 C_*x*_H_1−*x*_ + *x*CO_2_ → 2*x*CO + (1 − *x*/2) H_2_

To summarize, the addition of promoters enhances both the catalytic activity and durability of Ni–La_2_O_3_ through three different roles. Firstly, the addition of MgO or other alkali metals oxides to Ni–La_2_O_3_ not only enhance the basicity of the catalyst but also it can create surface oxygen ionic species that enhance the stability of La_2_O_2_CO_3_ phase which is liable for gasification of deposited coke, protecting Ni active centers from deactivation.^33,61^

The greater number of oxygen vacancies and the higher oxygen mobility enhances the resistance to coke formation.^62^ Furthermore, the incorporation of alkali metals and alkaline earth metals increase the density of lattice oxygen surface species which enhances the activation of methane C–H bond and thus foster the catalytic activity.^21^ Last but not least, the promoters enhance the surface alkalinity through increasing the number of basic sites which in turn enhance the activation of CO_2_ and facilitate the gasification of deposited carbon at the active sites.^63^ This was confirmed by the absence of carbon deposits in all spent catalysts except Ni–La_2_O_3_ owing to low basicity compared to Mg and other alkali metals.

## 4. Conclusions

Promoted and un-promoted Ni–La_2_O_3_ catalysts were successfully synthesized *via* one-step solution combustion route and their activity towards DRM was studied. All samples manifested high specific surface area and enhanced porosity owing to rapid ignition/combustion process and evolution of large amount of gases. In terms of DRM performance, all samples afforded high catalytic activity with CO_2_ and CH_4_ conversions higher than the equilibrium values at 700 °C. Promoted catalysts demonstrated enhanced catalytic activity and durability compared to un-promoted sample, which was ascribed to enhanced Lewis basicity. Particularly, Mg–Ni–La_2_O_3_ exhibited outstanding anti-coking capability compared to other samples. This can be assigned to two main factors. The first is the high surface area and high porosity compared to its counterparts, thereon, it can provide more active sites for DRM which enhances the catalytic activity. In addition, the incorporation of Mg afforded the increased number of basic sites while maintaining strong MSI compared to other promoters. This enhances the adsorption of CO_2_ and the supply of surface oxygen species, which inhibit the coke deposition and retard the catalyst deactivation *via* high temperature sintering. This is evidenced by comparing the percentages of adsorbed oxygen in promoted and unpromoted catalysts. In the unpromoted sample, the adsorbed oxygen constitutes 12.0% of the total oxygen, while in the promoted catalysts, the ratio of adsorbed oxygen ranges between 44–54% (see Fig. S3 and Table S1†). On the other hand, among all promoted catalysts, only Li–Ni–La_2_O_3_ revealed decay in long-term stability. This was assigned to the high temperature sintering endowed by weakened MSI.

## Conflicts of interest

There are no conflicts to declare.

## Supplementary Material

RA-011-D1RA05511A-s001
