# Supplementary material for: Solution combustion synthesis of Ni/La2O3 for dry reforming of methane: tuning the basicity via alkali and alkaline earth metal oxide promoters
Source: RSC Adv. 2021 Oct 15;11(53):33734–43. doi: 10.1039/d1ra05511a (PMC9042257; doi:10.1039/d1ra05511a)
Supplement: RA-011-D1RA05511A-s001 [file RA-011-D1RA05511A-s001.pdf]

Electronic Supplementary Material (ESI) for RSC Advances.
This journal is © The Royal Society of Chemistry 2021

Solution Combustion Synthesis of Ni/La_2O_3 Catalysts Promoted with Alkali and Alkaline Earth Metal Oxides for Dry Reforming of Methane

Yahia H. Ahmad,[a] Assem T. Mohamed,[a] Anand Kumar,[b] and Siham Y. Al-Qaradawi*,[a]

a. Department of Chemistry and Earth Sciences, College of Arts and Sciences, Qatar University, Doha 2713, Qatar

b. Department of Chemical Engineering, College of Engineering, Qatar University, Doha 2713, Qatar

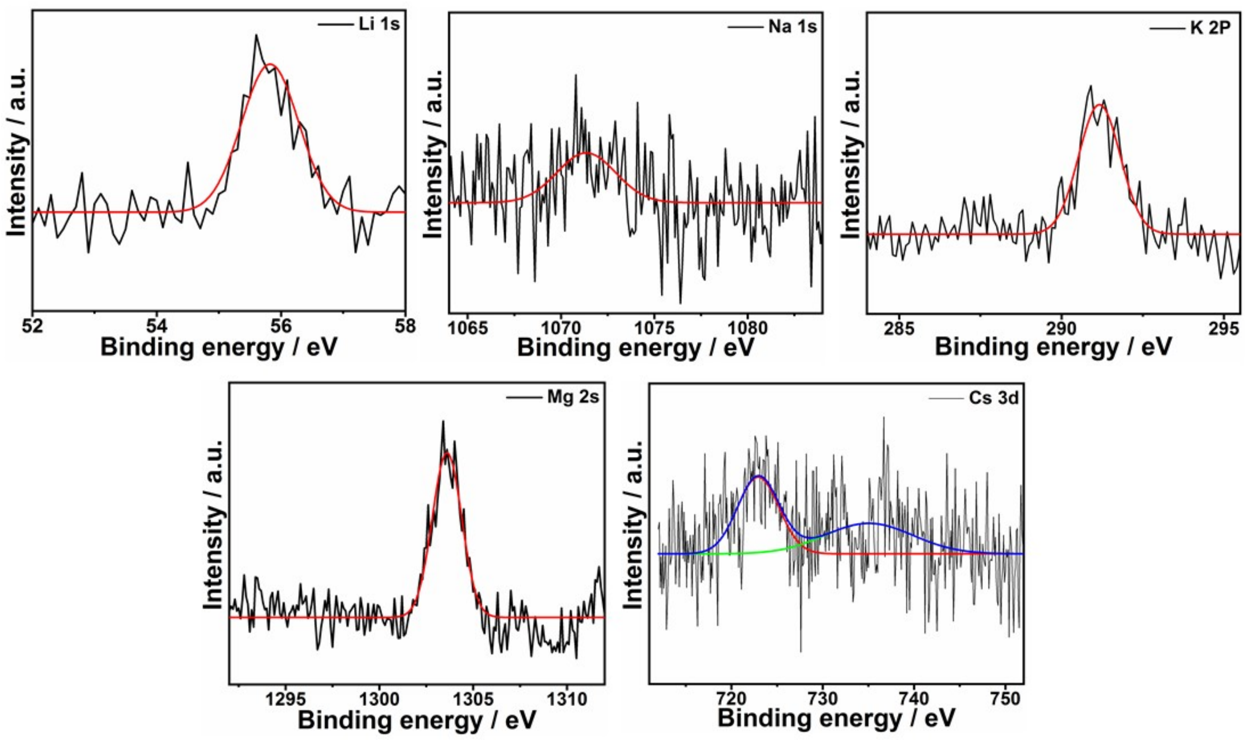

Figure S1. High resolution spectra of promoter species.

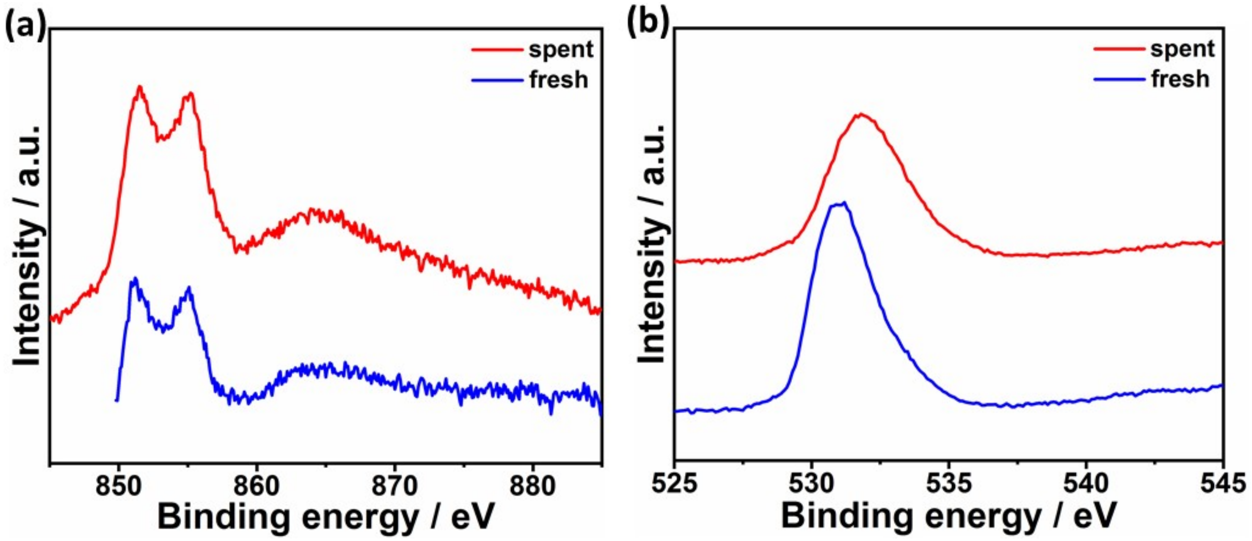

Figure S2. High resolution spectra of (a) Ni 2p and (b) O 1s in freshly reduced and spent Mg-Ni-La_2O_3.

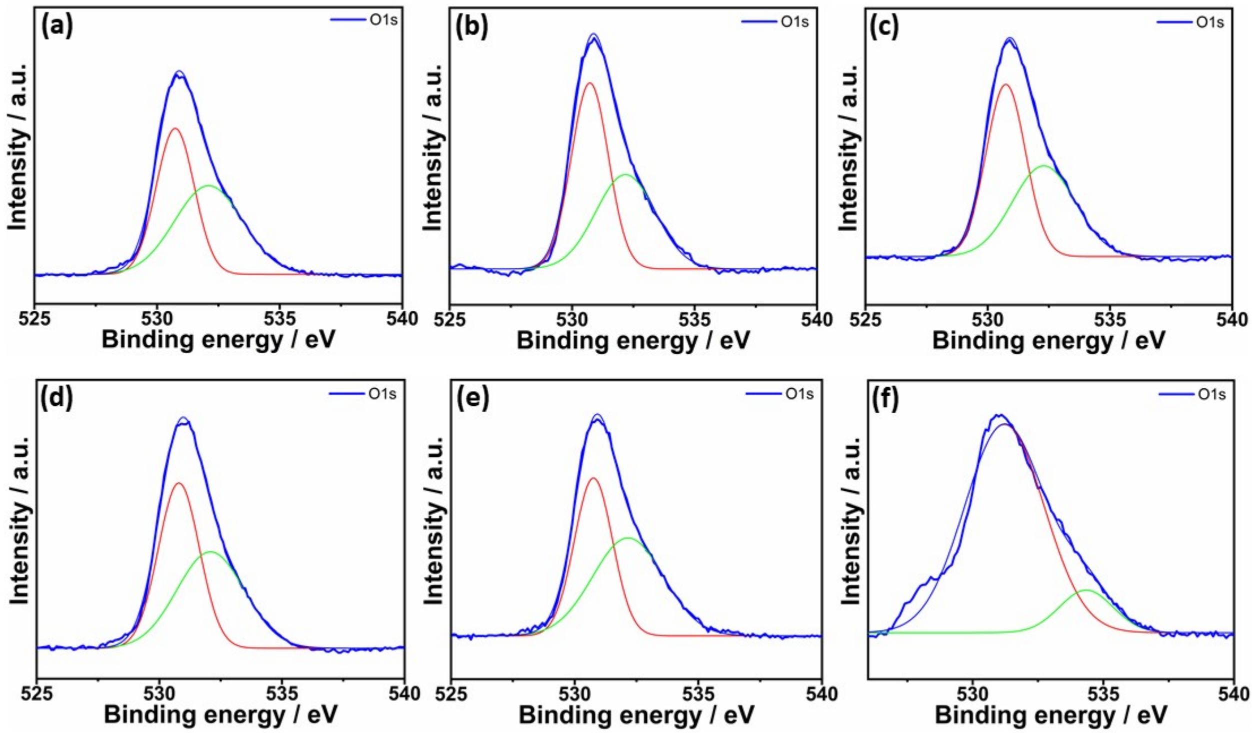

Figure S3. Deconvoluted O 1s spectra of reduced (a) Li-Ni-La_2O_3, (b) Na-Ni-La_2O_3, (c) K-Ni-La_2O_3, (d) Mg-Ni-La_2O_3, (e) Cs-Ni-La_2O_3, and (f) Ni-La_2O_3.

Table S1. Ratio of adsorbed oxygen species in reduced catalysts

Catalyst	Percentage of adsorbed oxygen (%)
Li-Ni-La_2O_3	52.0
Na-Ni-La_2O_3	44.8
K-Ni-La_2O_3	45.0
Mg-Ni-La_2O_3	48.9
Cs-Ni-La_2O_3	53.5
Ni-La_2O_3	12.0

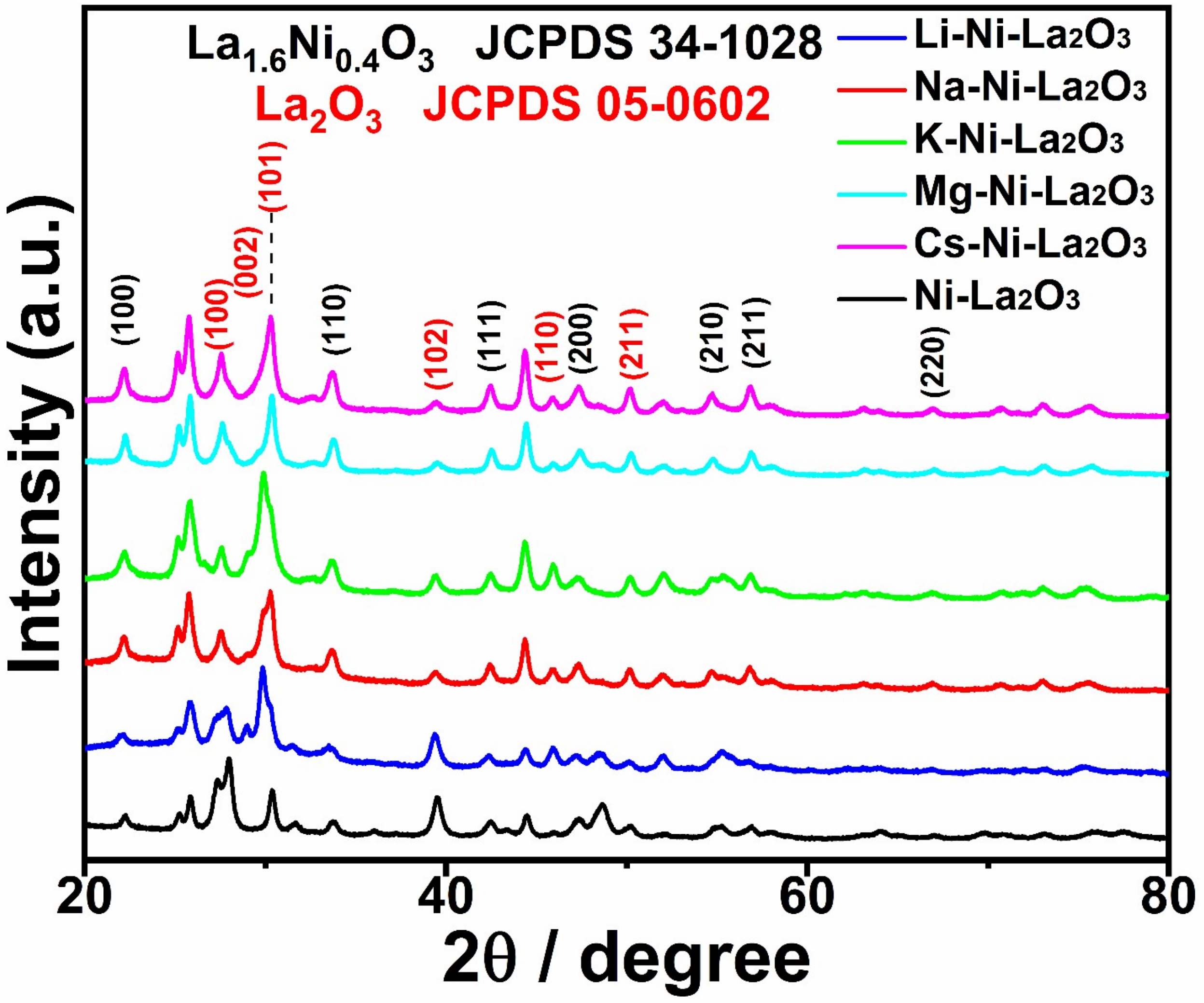

Figure S4. XRD patterns of as-prepared catalysts.
